# Effects of Kathon, a Chemical Used Widely as a Microbicide, on the Survival of Two Species of Mosquitoes

**DOI:** 10.3390/molecules26144177

**Published:** 2021-07-09

**Authors:** Wen-Ze He, Li-Long Pan, Wen-Hao Han, Shaaban Abd-Rabou, Shu-Sheng Liu, Xiao-Wei Wang

**Affiliations:** 1 Ministry of Agriculture Key Laboratory of Molecular Biology of Crop Pathogens and Insects, Institute of Insect Sciences, Zhejiang University, Hangzhou 310058, China; 12016107@zju.edu.cn (W.-Z.H.); panlilong@zju.edu.cn (L.-L.P.); wenhao_han@zju.edu.cn (W.-H.H.); shshliu@zju.edu.cn (S.-S.L.); 2 Egypt Agricultural Research Center, Plant Protection Research Institute, Giza 12311, Egypt; shaabanabdrabou59@yahoo.com

**Keywords:** Kathon, *Culex quinquefasciatus*, *Aedes albopictus*, larvicide

## Abstract

In recent decades, demands for novel insecticides against mosquitoes are soaring, yet candidate chemicals with desirable properties are limited. Kathon is a broad-spectrum isothiazolinone microbicide, but other applications remain uncharacterized. First, we treated larvae of *Culex quinquefasciatus* and *Aedes albopictus*, two major mosquito vectors of human viral diseases, with Kathon at 15 mg/L (a concentration considered safe in cosmetic and body care products), and at lower concentrations, and found that Kathon treatment resulted in high mortality of larvae. Second, sublethal concentration of Kathon can cause significantly prolonged larval development of *C. quinquefasciatus*. Third, we explored the effects of two constituents of Kathon, chloromethylisothiazolinone (CMIT) and methylisothiazolinone (MIT), on the survival of larvae, and found that CMIT was the major toxic component. Further, we explored the mechanisms of action of Kathon against insect cells and found that Kathon reduces cell viability and adenosine triphosphate production but promotes the release of lactate dehydrogenase in *Drosophila melanogaster* S2 cells. Our results indicate that Kathon is highly toxic to mosquito larvae, and we highlight its potential in the development of new larvicides for mosquito control.

## 1. Introduction

Mosquito-borne infectious diseases are a continuing threat to human health across the world and cause over one million deaths each year [1]. In recent decades, we have seen the re-emergence/emergence of epidemic diseases caused by many arboviruses such as Zika, chikungunya, west Nile virus and dengue virus [2,3]. Several mosquito species in the Culicidae are responsible for the spread of these viruses. For example, *Culex* mosquitoes, such as *C. quinquefasciatus*, are responsible for the transmission of west Nile virus, and *Aedes* mosquitoes, such as *A. aegypti* and *A. albopictus*, are major vectors of several diseases such as dengue and chikungunya [1,4]. Since the abundance of adult mosquito vectors is a direct, major determinant of the chance of disease spread among humans, disease control can be achieved through successful management of mosquito populations [2].

Currently, various strategies have been developed to control mosquitoes. For example, long-lasting insecticide impregnated nets and indoor residual spraying are highly effective in targeting indoor adult mosquitoes, thereby reducing the probability of pathogen transmission [2,5]. Mosquito repellants such as DEET (*N*,*N*-diethyl-*meta*-toluamide) are widely used and effective in protecting humans from mosquito bites [6]. Further, control of mosquitoes can be achieved by the suppression of larval population through management of aquatic habitats that are potential breeding sites [2,7]. To this end, application of larvicides has played a significant role [7]. While various insecticides have been harnessed in the control of mosquito larvae, development of novel insecticides remains critical due to the rapid emergence of insecticide resistance [2]. However, development of novel insecticides has been challenging due to the increasing costs of discovery and development [8]. Hence, alternative strategies, such as identifying and repurposing existing and widely used chemicals that are not currently used as insecticides, may accelerate the development of novel insecticides.

Kathon, a member of isothiazolinones that have been shown to be effective in suppressing the growth of various microorganisms such as bacteria, is a broad-spectrum microbicide for the preservation of a wide variety of household and industrial products, such as shampoos, liquid soaps, body washes and shower gels [9,10]. The upper limit of its concentration in cosmetic products is set as 15 mg/L (https://eur-lex.europa.eu/legal-content/EN/TXT/?uri=CELEX%3A32009R1223&qid=1607496617829, accessed on 9 December 2020) [11]. It exhibits excellent antimicrobial activity against bacteria, molds and yeasts [9,12]. Kathon is composed of 5-chloro-2-methyl-4-isothiazolin-3-one (chloromethylisothiazolinone, CMIT) and 2-methyl-4-isothiazolin-3-one (methylisothiazolinone, MIT) in a 3:1 ratio [12]. Previously, we found Kathon is insecticidal against several hemipteran insects [13]. Since the insecticidal activity of Kathon against mosquito larvae has not been explored, we sought to determine the effects of Kathon on the survival of the larvae of *C. quinquefasciatus* and *A. albopictus*, two major mosquito vectors of human diseases. In addition, we used a cell line of the model insect *Drosophila melanogaster* to explore the mechanisms of insecticidal action of Kathon.

## 2. Results

### 2.1. The Effects of Kathon on the Survival of C. quinquefasciatus Larvae

We first tested the effects of Kathon on the survival of each larval instar of *C. quinquefasciatus*. For each of the four instars, Kathon treatment caused mortality of *C. quinquefasciatus* larvae in a concentration-dependent manner (Table 1). At the lowest concentrations tested (1.88 mg/L), Kathon killed 53.80 ± 6.70%, 0.00 ± 0.00%, 70.76 ± 5.51% and 58.16 ± 2.08% of first, second, third and fourth instar larvae 24 h after treatment, respectively. Mortality increased with Kathon concentration and reached 96% for the first instar larvae at a concentration of 15.00 mg/L 24 h after treatment. Mortality increased for each of the instars from 24 h to 48 h after treatment and approached or reached 100% at 48 h (Table 1).

### 2.2. The Effects of Kathon on the Survival of A. albopictus Larvae

We then tested the effects of Kathon on the larval survival of another mosquito species, *A. albopictus*. Similar to the bioassays with *C. quinquefasciatus*, Kathon treatment killed larvae of each of the four instars of *A. albopictus* in a concentration-dependent manner (Table 2). Overall, Kathon treatment caused lower mortality of *A. albopictus* than that observed in *C. quinquefasciatus*. Similar to *C. quinquefasciatus*, only a small increase of larval mortality was observed from 24 h to 48 h after treatment (Table 2).

### 2.3. The Effects of Sublethal Concentrations of Kathon on the Larval Development Duration of C. quinquefasciatus

We then examined the sublethal effects of Kathon on mosquito larvae. Larvae of *C. quinquefasciatus* treated with sublethal concentrations of Kathon suffered low rates of mortality in each of the four instars tested (data not shown). However, their development duration was significantly extended in each of the four instars, and the extension significantly increased with increasing Kathon concentration in the third and fourth instars (Table 3).

### 2.4. The Effects of CMIT and MIT on the Survival of C. quinquefasciatus Larvae

To determine which constituents of Kathon were acting as larvicides, the two constituents were individually administrated to mosquito larvae; the results are presented in Table 4. When the fourth instar larvae of *C. quinquefasciatus* were treated with CMIT, they suffered significant levels of mortality, and the mortality increased with the concentration of CMIT, reaching 58% and 69% at 24 h and 48 h, respectively, following treatment with 11.25 mg/L of CMIT. In contrast, no mortality of the fourth instar larvae occurred at 24 h following treatment with 1.88 mg/L of MIT, and significant mortality occurred only at 48 h following treatment with a concentration of 3.75 mg/L of MIT, the highest concentration in combination with the longest time tested in this study. 

### 2.5. Effects of Kathon on Cell Viability, Adenosine Triphosphate (ATP) Production and Lactate Dehydrogenase Release of S2 Cells

**Figure 1:**
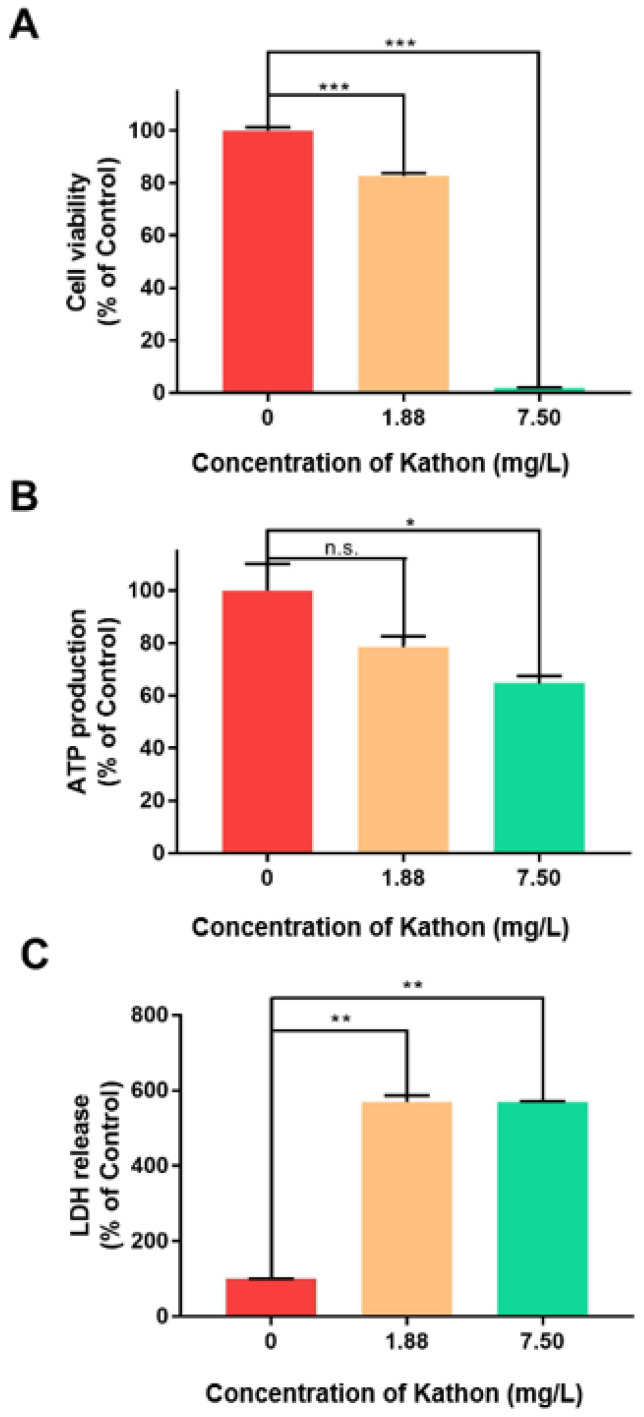
Effects of Kathon on cell viability (**A**), adenosine triphosphate (ATP) production (**B**) and lactate dehydrogenase release (**C**) of S2 cells. Cell viability was determined using the CCK8 assay. ATP production and level of lactate dehydrogenase (LDH) were measured using commercially available kits. All data are presented as mean ± standard error of mean (SEM). * stands for *p* < 0.05, ** stands for *p* < 0.01 and *** stands for *p* < 0.001 (independent *t*-test). n.s. stands for no significant difference.

To explore the mechanism of action of Kathon, *Drosophila melanogaster* S2 cells were treated with Kathon. Viability of S2 cells decreased following treatment with Kathon in a concentration-dependent manner; little viability was observed when the cells were treated with Kathon at 7.50 mg/L (Figure 1A). Similarly, ATP production of S2 cells significantly decreased following Kathon treatment, again in a concentration-dependent manner (Figure 1B). To examine cell membrane damage, we measured the lactate dehydrogenase (LDH) level in the cell culture medium, which indicates the extent of cell damage [14]. Kathon treatment substantially increased the level of LDH in the culture medium, indicating that the cell membrane was damaged when exposed to Kathon (Figure 1C).

## 3. Discussion and Conclusions

Many chemical insecticides have been used for mosquito control, including organophosphates and organochlorines [15]. These insecticides tend to be persistent, toxic to non-target organisms and are prone to the development of resistance by mosquitoes; these drawbacks have resulted in the banning of some organochlorines, such as DDT and most organophosphates, in many regions [16]. Yet, organophosphate temephos (commercially known as Abate) is still used widely as a larvicide in mosquito breeding sites due to its high and stable efficacy and additional low cost [17]. As concerns related to insecticide resistance and harm to human health increase regarding organophosphates, such as temephos, novel larvicides that are environmentally friendly, safe to humans and cost-effective are urgently needed [15,18]. 

In bioassays, Kathon causes high mortality of larvae of *C. quinquefasciatus* and *A. albopictus* when treated with this compound at 15 mg/L or lower concentrations (Table 1 and Table 2). Noticeably, over 50% mortality occurred when the larvae of *C. quinquefasciatus* were treated with a low concentration of Kathon at 1.88 mg/L (1.88 ppm). The data also show that exposure of *C. quinquefasciatus* larvae to sublethal concentrations of Kathon causes significant delays in development (Table 3). In addition, of the two constituents of Kathon, CMIT may play a dominant role in killing mosquito larvae (Table 4). Notably, when compared to some other novel insecticides such as essential oils, which are effective in the low ppm range, Kathon is of similar if not higher efficacy [19,20]. Unlike essential oils, which rapidly degrade in the environment, isothiazolinones such as Kathon are stable under ambient temperatures for at least one year [21,22]. Additionally, Kathon is bioactive over a wide pH range [9,10,12]. Its high efficacy and appreciable stability highlight the potential application of Kathon in mosquito control.

While Kathon has been reported to cause allergic dermatitis responses [23,24], risk analysis indicated that, in general, current use of Kathon is safe [25]. Kathon is known to rapidly degrade in living organisms upon oral administration and does not bio-accumulate in the environment [9]. Moreover, many in vivo studies, including subchronic, chronic, reproductive and developmental studies using laboratory animals, have shown that there is no sign of neurotoxicity [9]. The LD50 of Kathon via the oral route for mammals such as rats is lower than most organophosphates, and it is lower than or similar to most organochlorines (https://www.merckvetmanual.com/toxicology/insecticide-and-acaricide-organic-toxicity/organophosphates-toxicity, accessed on 26 June 2021) [9,26]. In view of the upper limit of 15 mg/L for the safe use of Kathon in leave-on and rinse-off cosmetics [11], as well as the fact that 15 mg/L and lower concentrations of Kathon can cause high mortality to the larvae of the two species of mosquitoes tested in this study (Table 1 and Table 2), we suggest that Kathon has the potential to be used in mosquito control, with limited risk to humans and the environment.

In addition to the toxicity enacted on mosquito larvae reported here, Kathon has been shown to be insecticidal against some hemipteran insects, including aphids and several whiteflies [13]; thus, we assume that Kathon may be toxic to a range of insects from different orders. Using the S2 cell lines of the model insect *Drosophila melanogaster* to investigate the insecticidal mechanisms of Kathon, we found that Kathon significantly reduces cell viability and ATP production, and increases lactate dehydrogenase release in S2 cells, which indicates that the cell membrane was damaged (Figure 1). The mechanism of action of isothiazolinone microbicides such as Kathon is complex, and it is believed to be a two-step process involving rapid growth inhibition followed by irreversible cell damage [9,12]. Upon isothiazolinone treatment, the activity of many specific enzymes, including dehydrogenases, and key physiological activities, such as respiration (oxygen consumption) and energy generation (ATP synthesis), is rapidly inhibited. The broad spectrum of isothiazolinones such as Kathon may be attributable to the fact that many of these key enzymes are present in many microorganisms. Therefore, we speculate that the mechanism of action of Kathon as a mosquito larvicide may be similar to that identified in microbicides. 

This original study reports on the mosquito larvicidal activity of Kathon. In the future, Kathon may be used in the management of aquatic habitats that are potential breeding sites to suppress larval population. To further explore the potential of Kathon in mosquito control, field experiments with various environmental settings should be conducted. Since Kathon has been reported to cause allergic dermatitis responses, risk analysis for the use of Kathon as mosquito larvicides should be conducted [23,24]. Likewise, as Kathon may be toxic to a range of insects of different orders (This study) [13], assessment of toxicity of this compound on beneficial organisms such as bees is required to explore its application in the field. In addition, the potential effects of Kathon on gut bacteria in mosquito larvae should be considered, as such effects may affect the efficacy of Kathon as a mosquito larvicide [27,28].

## 4. Materials and Methods

### 4.1. Chemicals

Kathon (3.0% in glycol) and MIT (9.5% in water) were purchased from Sigma-Aldrich. CMIT (1.5% in water) was purchased from Hubei Jusheng Technology Co., Ltd. (Hubei, China).

### 4.2. Mosquitoes

Two species of mosquito, *C. quinquefasciatus* and *A. albopictus*, were used in the bioassays. The colony of *C. quinquefasciatus* was established from a field collection in Foshan, China, in 1990 and has been maintained in the laboratory for 29 years (kindly provided by Professor Jianchu Mo, Institute of Insect Sciences, Zhejiang University). The colony of *A. albopictus* was established from a field collection in Shanghai, China, in 2000 and has been maintained in the laboratory for 20 years (kindly provided by Professor Sibao Wang, Shanghai Institutes for Biological Sciences, Chinese Academy of Sciences). The larvae of the two species were reared in plastic pots (5 L) filled with dechlorinated water and mouse fodder was supplied as food (0.2 g every three days). Prior to adult emergence, mosquito pupae were transferred to cloth cages (30 cm × 30 cm × 30 cm). Post emergence, adult mosquitoes were given 5% sucrose solution and blood meals were provided using laboratory mice. Rearing and experiments were conducted in climate-controlled chambers at 26 ± 2 °C, 60–70% relative humidity and 14/10 h light/dark cycles. Beakers (100 mL) filled with water were used for oviposition by *C. quinquefasciatus*, and Petri dishes (90 mm) containing wet filter papers were used for oviposition by *A. albopictus*.

### 4.3. Effects of Kathon on Mosquito Larval Survival 

For the bioassays with mosquito larvae, Kathon was first diluted with water to four concentrations, namely 15.00, 7.50, 3.75 and 1.88 mg/L. These concentrations were designed with the following considerations: (1) The regulation of the European Union on cosmetic products states that the upper limit of Kathon in leave-on and rinse-off cosmetics is 15mg/L (https://eur-lex.europa.eu/legal-content/EN/TXT/?uri=CELEX%3A32009R1223&qid=1607496617829, accessed on 9 December 2020) [11]. This upper limit of 15 mg/L was taken as the highest concentration to be tested. Additionally, (2) three lower concentrations were then designed by halving the concentration consecutively. Water was used as negative control. In the bioassays on *A. albopictus*, 10–17 larvae were used for each replicate. For the 1st, 3rd and 4th instar *C. quinquefasciatus* larvae, 7–20 larvae were used for each replicate, and 4–10 larvae were used for 2nd instar. The larvae were collected and placed into a beaker (100 mL) filled with Kathon solutions or water. Dead and live larvae were counted 24 and 48 h later. Larvae were considered dead when they did not move upon touch. For each of the concentration in a given instar, five replicates were conducted. 

### 4.4. Effects of Sublethal Concentrations of Kathon on the Larval Development Duration of C. quinquefasciatus

Following survival bioassays, we attempted to test the effects of sublethal concentrations of Kathon on the development duration of each of the four larval instars of *C. quinquefasciatus*. Two sublethal concentrations of LC10 and LC30 were estimated for each instar from the data of the above experiments. Water was used as negative control. In each replicate, 8–10 larvae of a given instar were collected and placed into a beaker (100 mL) filled with Kathon solution or water (control). Forty-eight hours later, larvae were transferred to dechlorinated water for rearing. The larvae were observed daily to record their development and were discarded when they had reached the next instar. For each of the concentrations in the test of a given instar, five replicates were conducted.

### 4.5. Effects of CMIT and MIT on the Survival of C. quinquefasciatus Larvae

Kathon is composed of CMIT and MIT in a 3:1 ratio [12]. When we found that Kathon at 7.50 and 15.00 mg/L could effectively kill larvae of *C. quinquefasciatus*, we attempted to determine the effect of the two constituents separately on the survival of the mosquito. CMIT was diluted with water to two concentrations, namely 11.25 and 5.63 mg/L, and MIT was diluted with water to two concentrations, namely 3.75 and 1.88 mg/L, which corresponded to one third of the concentrations of CMIT. In each replicate of a given concentration of either CMIT or MIT, 10–21 *C. quinquefasciatus* larvae of the 4th instar were placed into a beaker (100 mL) filled with Kathon solution and, 24 and 48 h later, live and dead larvae were counted. For each concentration, four replicates were conducted.

### 4.6. Effect on Insect Cells

#### 4.6.1. Cell Culture

*Drosophila melanogaster* S2 cells (Life Technologies, New Castle, Australia) were cultured in fresh, sterile filtered, Complete Schneider’s *Drosophila* media (CSD). CSD media was made by mixing Schneider’s Drosophila Medium with 10.0% *v/v* heat-inactivated fetal bovine serum and 1.0% *v/v* penicillin-streptomycin. Cells were passaged every three days.

#### 4.6.2. Cell Viability

Cell viability was determined using the CCK8 assay (Product A311, Vazyme, Nanjing, China), according to the manufacturer’s instructions. The CCK8 assay utilizes a tetrazolium salt 2-(4-iodophenyl)-3-(4-nitrophenyl)-5-(2,4-disulfophenyl)-2*H*-tetrazolium that produces a highly water-soluble formazan upon treatment by live cells [29]. Cells (10^5^) were seeded (100 μL CSD medium/well) onto 96-well plates and cultured overnight. Stock solutions (1.88 and 7.50 g/L) of Kathon were added to the medium to obtain the final concentrations of 1.88 and 7.50 mg/L, and water was used as control. The cells were incubated for 24 h. Subsequently, 10 μL of CCK8 solution was added to each well and the plate was incubated for 3 h at 37 °C. The absorbance was measured at 450 nm in SpectraMax iD5 Multi-Mode Microplate Reader (Molecular Devices, San Jose, CA, USA). Cell viability was expressed as the percentage of live cells/control cells.

#### 4.6.3. Adenosine Triphosphate (ATP) Assay

ATP production was measured using a commercially available kit (Product S0026, Beyotime Biotechnology, Nantong, China), according to the manufacturer’s protocols. Briefly, cells (10^5^) were seeded (2 mL CSD medium/well) in 12-well plates and cultured overnight. Stock solutions (1.88 and 7.50 g/L) of Kathon were added to the medium to obtain the final concentration of 1.88 and 7.50 mg/L, and water was used as control. The cells were further incubated for 24 h. Next, 200 μL lysis solution was added to each well and then medium in each well was collected and centrifuged at 12,000× *g* for 5 min. Twenty μL of the cell supernatant were collected and mixed with 100 μL of working solution and then transferred to wells of a 96-well plate. The luciferase signal was detected by a microplate luminometer (Molecular Devices, USA). ATP levels of the treated groups were calculated as normalized to those of the control group (100%).

#### 4.6.4. Lactate Dehydrogenase Assay

The level of lactate dehydrogenase (LDH) was determined using a commercially available LDH assay kit (Product C0016, Beyotime Biotechnology, Nantong, China), according to the manufacturer’s instructions. Cells (10^5^) were seeded (2 mL CSD medium/well) in 12-well plates and cultured overnight. Cells were then collected and washed once with PBS, and then fresh CSD medium was added. Next, stock solutions (1.88 and 7.50 g/L) of Kathon were added to the medium to obtain the final concentration of 1.88 and 7.50 mg/L, and water was used as control. The LDH reaction solution was added and then cells were further incubated for 30 min. At last, medium was collected and centrifuged at 400× *g* for 5 min. The supernatant (120 μL) was collected into 96-well plates for analysis of absorbance. The absorbance was measured at 490 nm by a SpectraMax iD5 Multi-Mode Microplate Reader (Molecular Devices, San Jose, CA, USA). The percentage release of LDH was determined relative to control (100%).

### 4.7. Statistical Analysis

All percentage data were arcsine square root transformed for statistical analysis and back-transformed for presentation. Comparisons of larval mortality between different treatments were performed using one-way analysis of variance (ANOVA), along with Fisher’s least significant difference tests. Comparisons of cell viability, ATP production and LDH level between control and treatment were performed using an independent *t*-test. The difference was considered significant when *p* < 0.05. All data are presented as mean ± standard error of mean (SEM). All statistical analysis was performed using SPSS 20.0.

## Figures and Tables

**Table 1:** Effects of different concentrations of Kathon on the mortality of larvae of *C. quinquefasciatus* in each instar.

Instars	Concentration (mg/L)	Mortality (%) ^†^	
		24 h	48 h
1	0.00	0.00 ± 0.00 ^d^	0.00 ± 0.00 ^c^
	1.88	53.80 ± 6.70 ^c^	68.40 ± 6.76 ^b^
	3.75	79.00 ± 1.18 ^b^	94.40 ± 3.92 ^a^
	7.50	83.80 ± 5.54 ^b^	100.00 ± 0.00 ^a^
	15.00	95.80 ± 2.58 ^a^	97.60 ± 2.40 ^a^
2	0.00	0.00 ± 0.00 ^d^	0.00 ± 0.00 ^d^
	1.88	0.00 ± 0.00 ^d^	0.00 ± 0.00 ^d^
	3.75	31.70 ± 8.38 ^c^	34.63 ± 9.17 ^c^
	7.50	66.68 ± 3.74 ^b^	68.58 ± 3.53 ^b^
	15.00	100.00 ± 0.00 ^a^	100.00 ± 0.00 ^a^
3	0.00	0.00 ± 0.00 ^e^	0.00 ± 0.00 ^d^
	1.88	70.76 ± 5.51 ^d^	62.62 ± 3.80 ^c^
	3.75	83.85 ± 1.52 ^c^	81.40 ± 3.54 ^b^
	7.50	94.43 ± 1.69 ^b^	100.00 ± 0.00 ^a^
	15.00	100.00 ± 0.00 ^a^	100.00 ± 0.00 ^a^
4	0.00	0.00 ± 0.00 ^d^	0.00 ± 0.00 ^d^
	1.88	58.16 ± 2.08 ^c^	73.41 ± 1.65 ^c^
	3.75	59.86 ± 2.08 ^c^	85.98 ± 1.42 ^b^
	7.50	90.53 ± 1.09 ^b^	100.00 ± 0.00 ^a^
	15.00	100.00 ± 0.00 ^a^	100.00 ± 0.00 ^a^

^†^ In these columns, the mortalities of mosquito larvae were compared among different concentrations for each combination of instar and time point. Means not followed by the same letter differ significantly at *p* < 0.05 (LSD test).

**Table 2:** Effects of different concentrations of Kathon on the mortality of larvae of *A. albopictus* in each instar.

Instars	Concentration (mg/L)	Mortality (%) ^†^	
		24 h	48 h
1	0.00	0.00 ± 0.00 ^e^	0.00 ± 0.00 ^e^
	1.88	18.64 ± 10.79 ^d^	32.26 ± 3.02 ^d^
	3.75	37.37 ± 2.01 ^c^	42.85 ± 2.24 ^c^
	7.50	71.22 ± 0.51 ^b^	84.37 ± 3.19 ^b^
	15.00	90.56 ± 1.58 ^a^	92.35 ± 2.09 ^a^
2	0.00	0.00 ± 0.00 ^e^	0.00 ± 0.00 ^e^
	1.88	15.81 ± 9.13 ^d^	15.81 ± 9.13 ^d^
	3.75	30.57 ± 0.66 ^c^	36.83 ± 3.54 ^c^
	7.50	70.66 ± 1.86 ^b^	72.81 ± 1.05 ^b^
	15.00	89.01 ± 1.11 ^a^	91.31 ± 1.80 ^a^
3	0.00	0.00 ± 0.00 ^d^	0.00 ± 0.00 ^e^
	1.88	15.81 ± 9.13 ^c^	15.81 ± 9.13 ^d^
	3.75	22.66 ± 7.57 ^c^	32.87 ± 2.71 ^c^
	7.50	49.75 ± 2.90 ^b^	52.26 ± 2.51 ^b^
	15.00	79.44 ± 3.16 ^a^	84.23 ± 3.01 ^a^
4	0.00	0.00 ± 0.00 ^d^	0.00 ± 0.00 ^d^
	1.88	0.00 ± 0.00 ^d^	0.00 ± 0.00 ^d^
	3.75	34.90 ± 3.28 ^c^	34.90 ± 3.28 ^c^
	7.50	43.96 ± 4.75 ^b^	47.23 ± 2.51 ^b^
	15.00	85.00 ± 2.86 ^a^	89.35 ± 2.29 ^a^

^†^ In these columns, the mortalities of mosquito larvae were compared among different concentrations for each combination of instar and time point. Means not followed by the same letter differ significantly at *p* < 0.05 (LSD test).

**Table 3:** Effects of sublethal concentrations of Kathon on larval development duration of *C. quinquefasciatus*.

Instars	Concentration (mg/L)	Development Time (Days) ^†^
1	0.00	3.78 ± 0.05 ^b^
	0.57	4.41 ± 0.13 ^a^
	0.98	4.62 ± 0.16 ^a^
2	0.00	3.77 ± 0.16 ^b^
	3.90	4.12 ± 0.21 ^ab^
	5.52	4.35 ± 0.23 ^a^
3	0.00	4.09 ± 0.09 ^c^
	0.65	4.53 ± 0.12 ^b^
	1.13	4.88 ± 0.05 ^a^
4	0.00	7.29 ± 0.22 ^c^
	0.47	8.15 ± 0.21 ^b^
	1.10	8.79 ± 0.17 ^a^

^†^ In this column, the development times were compared among different concentrations for each instar. Means not followed by the same letter differ significantly at *p* < 0.05 (LSD test).

**Table 4:** Effects of different concentrations of CMIT and MIT on the survival of 4th instar larvae of *C. quinquefasciatus*.

Treatment	Concentration (mg/L)	Mortality (%) ^†^	
		24 h	48 h
CMIT	0.00	0.00 ± 0.00 ^c^	0.00 ± 0.00 ^c^
	5.63	14.77 ± 3.02 ^b^	46.59 ± 3.41 ^b^
	11.25	57.50 ± 5.13 ^a^	69.32 ± 3.86 ^a^
MIT	0.00	0.00 ± 0.00 ^a^	0.00 ± 0.00 ^b^
	1.88	0.00 ± 0.00 ^a^	0.00 ± 0.00 ^b^
	3.75	0.00 ± 0.00 ^a^	28.44 ± 2.77 ^a^

^†^ In these columns, the mortalities of mosquito larvae were compared among different concentrations for each combination of chemical and time point. Means not followed by the same letter differ significantly at *p* < 0.05 (LSD test).

## Data Availability

Data in this study are available upon request.

## Sample Availability

Samples of the compounds Kathon, CMIT and MIT are available from the authors upon reasonable request.
